# Book Notices

**Published:** 1867-03

**Authors:** 


### BOOK NOTICES.

*The Science and Practice of Medicine.* By WILLIAM AITKEN, M.D., Edin., Professor of Pathology in the Army Medical School, and formerly Demonstrator of Anatomy in the University of Glasgow, etc. In Two Volumes. Vol. I. 8vo, pp. 955. From the Fourth London Edition, with Additions by MEREDITH CLYMER, M.D., Late Professor of the Practice of Medicine in the University of New York, etc. Philadelphia: Lindsay & Blakiston. 1866.

Here we have the first volume of the American reprint of one of the greatest works of the time. It is not a hand-book for the use of students merely, but a work of almost encylopædic dimensions, for the information of physicians. It may well be considered the highest expression of medical thought in England at the present date. The author is richly gifted with those traits which characterize the best teachers and the wisest men. To the conservatism of age and experience, he unites the enthusiasm and the acuteness of youth. Refusing to surrender the acquisitions of past generations, he treats them, however, as capital which shall yield a hundred-fold interest under his care. His book, therefore, serves as well to stimulate the efforts of the studious, as to repress the sneers of those who would undervalue the labors of the past. An example of this may be found in his remarks upon the subject of blood-letting, while, with Alison, he believes in the good effects of bleeding in the treatment of inflammation, his opinions, with regard to the use of so powerful a remedy, are greatly modified by the teachings of Bennett.

The introductory chapters, which treat of the principles of medicine, are replete with valuable matter. The use of the thermometer in diagnosis is fully explained for the first time in any systematic work. That which, for want of a better term, may be called the *chemistry of disease*, here receives the attention which the advance of physiological chemistry demands. The progress of sanitary science is duly noted, together with many pregnant topics which relate to the diminution of disease and the prolonging of life.

From general principles, the author proceeds to the consideration of general diseases. The present volume treats of zymotic diseases. The second volume, which is soon to appear, will treat of constitutional diseases and inflammations. Commencing with small-pox, we are treated to a full discussion of the eruptive fevers. Among the symptoms of each, the temperature of the patient is made prominent, by the use of illustrative tables, such as our readers were made acquainted with by Dr. Seguin's article in our May number, last year. Passing to the continued fevers, Dr. Aitken recants his former belief, and recognizes the specific distinctions between typhus and typhoid fever. The thermometric differences between these two fevers are sufficient to disprove the notion of their identity, while affording the means of differential diagnosis at an earlier stage of the disease than has been hitherto possible. In typhoid fever, the temperature rises gradually from the normal standard, by regular daily increments of about 2° Fahr., until the height of the fever (103° to 104° Fahr.) is reached on the fourth day. The temperature rarely exceeds this point, but defervescence does not often occur before the fourth week, and is then gradual. In typhus, the temperature is greater—102° or 103° Fahr. on the first day—and it reaches 107° Fahr. on the fourth day. Defervescence occurs suddenly during the third week. "When the temperature on the first or second day reaches to 104°, or where, in a child or in an adult, the evening temperature between the fourth and sixth days does not rise to 103°, where in the second half of the first week there is considerable abatement of the evening temperature, we have in such cases certainly *not to do with typhoid fever.*"

The remarks of our author upon the origin and propagation of zymotic diseases are valuable. Granting the fact that we cannot penetrate to the primary source of a zymotic disease, he doubts the doctrine of an origin *de novo* for diseases of this class. "Plants and animals—likewise, at least, two diseases, namely, *syphilis* and *small-pox*—are certainly now known to propagate only by the law of continuous succession, whatever may have been their primary source." Filth, foul air, and debility serve as predisposing causes, but are never the first cause of a zymotic disease. Living germs transported from the subject of disease and received into the blood of susceptible individuals constitute the only exciting cause of diseases of this class. As small-pox begets small-pox, typhoid fever begets typhoid fever, never changing its type nor appearing as typhus or any other fever. The idea that from dysentery, for example, in some way, under certain conditions, a specific disease like typhus may arise is absurd. "To urge such an hypothesis is to ignore all evidence, and to render the progress of medical science impossible." Such a view of the subject adds greatly to its simplicity.

We might continue to multiply extracts from this great work, were it necessary thus to acquaint our readers with its value. We hope that it will be thoroughly studied by every member of the profession, for this is something more than a book of symptoms and treatment. Its pages discuss the totality of disease instead of being confined to the incidents and accidents of mere bedside observation. For this reason, these volumes will ever be prized by all who regard *preventio* as something better than *cure*, by all who value the preservation of public health as well as the relief of Individual suffering.

The publishers have done their work admirably. The book is printed from clear type, on good paper, in a style that almost matches the original English edition.

For sale by W. B. Keen & Co., Chicago. L.

